# Predicting coarse-grained semantic features in language comprehension: evidence from ERP representational similarity analysis and Chinese classifier

**DOI:** 10.1093/cercor/bhad116

**Published:** 2023-04-04

**Authors:** Zirui Huang, Chen Feng, Qingqing Qu

**Affiliations:** Key Laboratory of Behavioral Science, Institute of Psychology, Chinese Academy of Sciences, Beijing 100101, China; Faculty of Linguistics, Philology and Phonetics, University of Oxford, Oxford OX1 2HG, United Kingdom; Key Laboratory of Behavioral Science, Institute of Psychology, Chinese Academy of Sciences, Beijing 100101, China; Department of Psychology, University of Chinese Academy of Sciences, Beijing 100049, China; Key Laboratory of Behavioral Science, Institute of Psychology, Chinese Academy of Sciences, Beijing 100101, China; Department of Psychology, University of Chinese Academy of Sciences, Beijing 100049, China

**Keywords:** semantic prediction, pre-activation of semantic features, Chinese classifier, EEG, representational similarity analysis

## Abstract

Existing studies demonstrate that comprehenders can predict semantic information during language comprehension. Most evidence comes from a highly constraining context, in which a specific word is likely to be predicted. One question that has been investigated less is whether prediction can occur when prior context is less constraining for predicting specific words. Here, we aim to address this issue by examining the prediction of animacy features in low-constraining context, using electroencephalography (EEG), in combination with representational similarity analysis (RSA). In Chinese, a classifier follows a numeral and precedes a noun, and classifiers constrain animacy features of upcoming nouns. In the task, native Chinese Mandarin speakers were presented with either animate-constraining or inanimate-constraining classifiers followed by congruent or incongruent nouns. EEG amplitude analysis revealed an N400 effect for incongruent conditions, reflecting the difficulty of semantic integration when an incompatible noun is encountered. Critically, we quantified the similarity between patterns of neural activity following the classifiers. RSA results revealed that the similarity between patterns of neural activity following animate-constraining classifiers was greater than following inanimate-constraining classifiers, before the presentation of the nouns, reflecting pre-activation of animacy features of nouns. These findings provide evidence for the prediction of coarse-grained semantic feature of upcoming words.

## Introduction

Probabilistic prediction is hypothesized to be an important computational principle underlying language comprehension (Federmeier 2007; Huettig 2015; Kuperberg and Jaeger 2016; Pickering and Gambi 2018; Pulvermüller and Grisoni 2020). A growing number of studies have demonstrated that people are able to make linguistic predictions at various representational levels including semantic (Altmann and Kamide 1999; Federmeier and Kutas 1999; Lau et al. 2013; Wang et al. 2018, 2020), phonological (DeLong et al. 2005; Vissers et al. 2006; Li et al. 2022), written form (Laszlo and Federmeier 2009; Kim and Lai 2012), and morphosyntactic (Van Berkum et al. 2005; Dikker et al. 2009, 2010) features of words. One question that has been investigated less is whether prediction can occur when prior context is less constraining for predicting specific words but can elicit the prediction of more general information. Most evidence comes from sentences with highly constraining context, in which a specific word is likely to be predicted. Here, we aim to address this issue by examining the prediction of semantic features in low-constraining context, using electroencephalography (EEG), in combination with representational similarity analysis (RSA).

A large group of studies support (not necessarily demonstrate) that people are able to predict semantic information of upcoming words. In a classic event-related potential (ERP) study by Federmeier and Kutas (1999), participants were instructed to read pairs of sentences (e.g. “They wanted to make the hotel look more like a tropical resort. So along the driveway, they planted rows of…”). The following word was either a predictable word (palms), an unpredictable word, but from the same semantic category (pines), or an unpredictable word from a different semantic category (tulips). They found that unpredictable words from the same category (pines) elicited smaller N400s compared to the unpredictable word from an unrelated category (tulips). The N400 effect varied as a function of the cloze probability of words, but not the plausibility of sentences, suggesting the effect reflects prediction-related pre-activation rather than integration of the target word with preceding context. Until recently, pre-stimulus predictive brain activity (i.e. prediction potential) was discovered (Grisoni et al. 2016, 2017, 2021; León-Cabrera et al. 2017; see Pulvermüller and Grisoni 2020, for a review). These studies have reported that high-constraining context induced anticipatory brain activity preceding the expected words, and the size of the anticipatory brain activity is correlated with the predictability of the expected word (see Grisoni et al. 2021). Converging evidence for semantic prediction comes from anticipatory eye movements toward objects before the expressions that refer to these objects. For instance, in a visual world task, Kamide et al. (2003) manipulated the agent of an action, and reported that when the preceding context was “the man will ride …”, listeners predictively looked at “motorbike” more than “carousel” before the presentation of the following nouns. The findings suggest that comprehenders use world knowledge about plausible actions by agents to predict semantic information of plausible actions.

Most work concerns the prediction of a specific word in a highly constraining context (Wicha et al. 2004; Ito et al. 2018; Wang et al. 2018; Li et al. 2022). It is less clear whether prediction can occur when prior context is less constraining for predicting specific words but can elicit the prediction of more general information that characterize a group of multiple words. Investigating whether comprehenders are only able to predict specific words at the fine-grained level or are also able to predict upcoming information at a coarse-grained level is theoretically important, because contexts that predict specific words (e.g. “I like coffee with sugar and ...”) are not frequent, and most words in natural language are not highly predictable on the basis of prior contexts or knowledge (e.g. I'd like to drink...) (Luke and Christianson 2016).

In the present study, we adopted the constraints of the classifier system in Mandarin Chinese, to test whether comprehenders can utilize the semantic constraints of classifiers to predict animacy feature of upcoming nouns. Chinese is a numeral classifier language, in which a classifier is obligatory between a numeral and a noun when a noun is modified by a numeral (e.g. one, two), a demonstrative (e.g. this, that), or a quantifier (e.g. a few) (Li and Thompson 1989), which contrasts to English where a noun immediately follows a numeral (e.g. one person). In other words, a classifier follows a numeral and precedes a noun in Chinese (e.g.  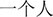, one “ge4” person). Chinese speakers need to select a proper classifier from 174 classifiers (c.f., Huang et al. 1997) based on semantic features of the accompanying noun. Therefore, a classifier can constrain semantic features of its following noun, such as animacy, shape, size, etc. Although the mapping between a classifier and a noun can be to some degree arbitrary so that a classifier can modify different nouns, and a noun can be preceded by various classifiers, some Chinese classifiers are unambiguous in specifying animacy features of nouns, therefore, constraining the animacy of following nouns. Some classifiers go with inanimate nouns only (e.g.  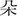,/duo/, classifying flowers) whereas some classifiers modify animate nouns (e.g.  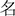 ,/ming/, classifying human noun, such as teacher, doctor etc.).

The classifier-noun association, combined with the violation paradigm where sentences are presented with classifier-noun mismatch errors, is commonly used to investigate semantic integration processes during Chinese sentence comprehension. A typical finding is that the classifier-noun mismatch elicits N400 effects on the noun, with larger negativity for mismatch trials, which reflects the difficulty of integrating the lexical semantics into the representation at the higher level (e.g. Zhou et al. 2010; Zhang et al. 2012). The regularity of the classifier-noun agreement, combined with ERPs, can provide efficient means to examine the effects of prediction during language comprehension. In one of few studies concerning semantic prediction in Chinese, Kwon et al. (2017) manipulated whether a classifier embedded in a sentence matched or mismatched an upcoming expected noun. Kwon et al. replicated the well-established N400 effect, with enhanced N400 amplitude to unexpected nouns. The N400 was also evident as early as the preceding classifier, suggesting the pre-activation of semantic features of nouns. However, the effect on classifiers may reflect integration of the classifier with the preceding context, rather than pre-activation of the noun (also see Szewczyk and Schriefers 2013). It is very difficult (if not impossible) to distinguish prediction from integration and demonstrate evidence that is compatible with prediction but not integration, by analyzing ERPs after encountering a target word, using the traditional ERP amplitude analysis.

In addition to ERP amplitude analysis, predictive behavior in language comprehension has recently been investigated using RSA (Kriegeskorte et al. 2008), i.e. analyzing similarity among patterns of neural activity, before or after encountering the target word. The basic assumption of RSA is that similarities between items can elicit similarities in patterns of brain activity. Wang et al. (2018) adopted RSA combined with magnetoencephalography (MEG) to examine whether comprehenders can predict specific words when they read highly constraining sentences. Wang et al. quantified the similarity between patterns of brain activity and found that the patterns of neural activity were more similar when the same words were predicted than when different words were predicted, critically before the onset of the predicted words. The results demonstrate that the prediction of specific words is associated with unique patterns of neural activity. In another study using RSA, Hubbard and Federmeier (2021) reanalyzed the previous EEG sentence reading study (Federmeier et al. 2007), and compared EEG activity similarity patterns elicited by sentence final words to patterns from the preceding words of the sentence. The logic is that if pre-final words elicit the pre-activation of features of the final word in constraining sentences, then some aspects of the neural representation of the final word should appear during the processing of the pre-final word, thus producing greater similarity between pre-final and final words. Measuring pattern similarity of the sentence final word and words prior to the pre-final word revealed that neural similarity with the final word was increased following the processing of only the pre-final word. The effect was not observed in earlier words and the increase was modulated by both final word expectancy and sentence constraint. These findings demonstrate a precisely timed semantic prediction.

In more recent work, Wang et al. (2020) used RSA to detect neural patterns for the prediction of animacy features of upcoming nouns when sentence contexts only constrain coarse-grained semantic animacy feature as opposed to a specific word. Semantic animacy of nouns were constrained by verbs (e.g. “caution” constrains for animate nouns; “unfold” constrains for inanimate nouns). RSA revealed that before the onset of nouns, patterns of neural activity were more similar following animate-constraining verbs than following inanimate-constraining verbs, providing evidence for the prediction of coarse-grained semantic features that goes beyond the prediction of individual words. One caveat with this type of design is that neural similarity may not reflect the similarity in the meanings of the predicted words but rather the similarity of the meanings of the preceding context, which is bound to correlate with the predicted target word.

In the present study, we reduce prior context and directly compare the EEG patterns after the presentation of classifiers without any additional constraining inputs. The goal is thus to investigate whether comprehenders can use the constraints provided by classifiers to predict semantic features associated with the animacy of upcoming nouns. We focus on the similarity between patterns of brain activity following the classifiers until just before the presentation of the nouns, although we analyze the similarity value after the presentation of nouns. Because animate entities share more strongly associative semantic features than inanimate entities, which have more distinctive features (McRae et al. 1997; Daniele Zannino et al. 2006), the brain activity patterns between word pairs should be more similar among animate nouns than inanimate nouns during the presentation of nouns. Critically, the question is whether the difference in similarity occurs before the presentation of nouns. If comprehenders predict the animacy of upcoming nouns, the similarity between patterns of neural activity should be greater following animate-constraining classifiers than following inanimate-constraining classifiers.

## Materials and method

### Participants

In total, 29 native speakers of Mandarin Chinese who were resident in Beijing participated in the ERP experiment. Sample size was determined by recent EEG/MEG RSA studies of language prediction (Hubbard and Federmeier, 2021; Wang et al. 2018). EEG data from four participants was excluded for data analysis due to high percentage of rejected trials (˃50%) or an empty data set for at least one classifier after data preprocessing, and thus 25 participants were included for ERP and RSA analyses. All participants had normal or correct-to-normal vision and no history of language disorders. They were given informed consent and paid ~$20. The study was approved by the Institutional Review Board of the Institute of Psychology, Chinese Academy of Sciences.

### Materials and design

A total of 12 classifiers were used, and each classifier was combined with 10 nouns to form 120 semantically plausible classifier-noun phrases. Among the 12 classifiers, six of them were animate-constraining classifiers that can only modify animate nouns. Of these, three were human-modifying classifiers and three animal-modifying classifiers; the other six classifiers were inanimate-constraining classifiers including three natural-object-modifying classifiers and three artifact-product-modifying classifiers. We have assessed the cloze probability of the classifiers using the cloze test. A group of 25 Chinese native speakers who did not take part in the EEG experiment were presented with incomplete phrase (i.e. Numeral + Classifier) and were asked to complete fragments with the most likely ending. Classifier constraint was measured as the proportion of participants who gave the most frequent word. Overall, the cloze probability of the classifiers was low (25.7% ± 11.3%), indicating that classifiers used in our study do not produce specific word predictions. Moreover, cloze probability was matched between animate-constraining classifiers (animate: 26.0% ± 7.0%) and inanimate-constraining classifiers (inanimate: 25.3% ± 1.6%), *P* = 0.934. Besides, these classifiers can highly constrain animacy of nouns as expected (probability of animate nouns following animate-constraining classifiers: 93.3%, probability of inanimate nouns following inanimate-constraining classifiers: 97.3%).

Each of 12 classifiers was recombined with incongruent nouns to form 120 semantically implausible classifier-noun phrases, including 60 semantically incongruent but animacy-matched trials (i.e. Incongruent, Animacy-Match), in which classifiers were re-paired with nouns from different subgroups (e.g. human classifiers were re-paired with animal nouns; artifact-product classifiers were paired with natural object nouns) and 60 semantically incongruent AND animacy-mismatched trials (i.e. Incongruent, Animacy-mismatch), in which classifiers were paired with nouns from different animacy groups (e.g. human classifiers were recombined with natural object nouns; artifact-product classifiers were recombined with animal nouns), see Table 1 for examples. The phrases were more implausible in the *Incongruent, Animacy-mismatch* compared to the *Incongruent, Animacy-Match* condition, due to the additional mismatch in animacy. That is, there was a graded difference in the degree of meaning congruence of the nouns and their classifiers across the three conditions. In total, 240 combinations of “classifier + noun” were used in the study. Each participant was presented with three blocks of 80 trials with the 40 congruent and 40 incongruent classifier-noun combinations in a randomized order.

**Table 1:** Experimental conditions and examples of stimuli.

Conditions	Animate-constraining classifier (CL)	Inanimate-constraining classifier (CL)
Congruent	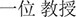 one CL_human_ professor	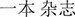 one CL_book_ magazine
Incongruent, Animacy-Match	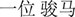 one CL_human_ horse	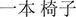 one CL_book_ chair
Incongruent, Animacy-Mismatch	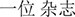 one CL_human_ magazine	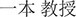 one CL_book_ professor

#### Quantifying the semantic and lexical similarity of the animate- versus inanimate-constraining classifiers

**Fig. 1:**
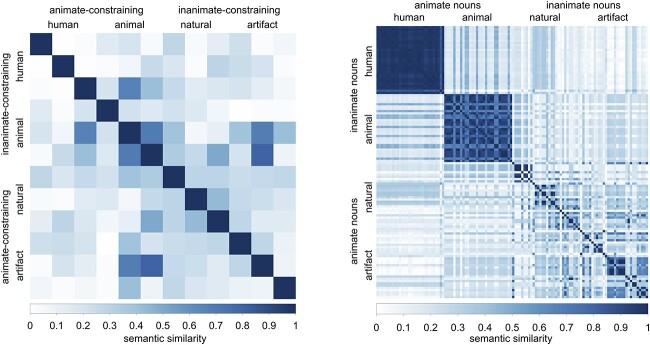
Pairwise Wu and Palmer similarity values for classifiers (A) and nouns (B). Similarity values are ranging from 0 to 1, with 0 indicating no similarity and 1 indicating identical semantics. (A) Shows a 12 by 12 symmetric semantic similarity matrix with six animate-constraining classifiers (three human classifiers and three animal classifiers) and six inanimate classifiers (three natural object classifiers and three artifact classifiers). (B) Shows a 120 by 120 semantic similarity matrix of nouns that are semantically matched with each classifier (each classifier is paired with 10 nouns), which confirmed the animacy constraint of classifiers.

Our aim was to measure the neural activity elicited by the pre-activation of animacy feature of the upcoming noun. Therefore, we focused on activity following the onset of the classifier until just before the onset of subsequent nouns. To make sure that any difference in the representational similarity of ERPs before the onset of nouns indeed reflected the pre-activation of the upcoming nouns rather than similarity associated within the animate- versus inanimate-constraining classifiers, we verified that the two groups of classifiers matched on several key properties relevant to visual and linguistic processing. These properties included semantic similarity, visual complexity and word frequency. To quantify semantic similarity between word pairs among the animate- versus inanimate-constraining classifiers, we used HowNet, an online database that provide calculations of inter-conceptual and inter-attribute relationships of Chinese lexicons (Dong et al. 2010). Semantic similarity values for all possible classifier pairs were measured via a path-based approach by Wu and Palmer (1994). These pairwise Wu-Palmer semantic similarity values in a 12 by 12 matrix are presented in Fig. 1A. The mean semantic similarity values for the animate- versus the inanimate-classifiers showed no difference (*t* = 0.15, *P* = 0.88). We also verified that other aspects of lexical similarity (i.e. visual complexity and word frequency) associated with the animate- versus inanimate-constraining classifiers were matched. In order to do that, we extracted visual complexity of Chinese characters of classifiers (the number of strokes) and word frequency (based on the Chinese Linguistic Data Consortium norms, 2013). The similarity values of visual complexity and word frequency were measured by the absolute difference for each possible pair of classifiers. Statistical analysis showed that visual complexity (*t* = −0.2, *P* = 0.84) and word frequency (*t* = 1.18, *P* = 0.26) were matched between the animate- and the inanimate- classifiers.

#### Quantifying the semantic and lexical similarity of the animate versus inanimate nouns

The experimental hypothesis rested on the assumption that animate nouns constrained for by their classifiers would be more semantically similar to each other than inanimate nouns. We used the same approach as described above to verify this assumption: We calculated the semantic similarity values between all possible pairs of nouns within the animate-constraining and inanimate-constraining conditions. As shown in Fig. 1B, the mean semantic similarity within the animate set was indeed greater than that within the inanimate set (*t* = 2.24, *P* = 0.03). Moreover, it is important to confirm that any differences in neural similarity produced by predicted animate and inanimate nouns were not generated by differences in similarity of lexical properties. Statistical analysis showed that visual complexity and word frequency were matched between animate versus inanimate nouns (visual complexity: *t* = 0.48, *P* = 0.63; word frequency: *t* = 1.14, *P* = 0.26).

### Procedure

The experiment was conducted using E-Prime software. Participants were first instructed that they would see the classifier-noun phrases presented on the computer screen and their task was to judge whether the classifier-noun phrase was semantically plausible or not by pressing a designated keyboard button. Each trial started with an asterisk signal for 500 ms, followed by a presentation of a numeral and a classifier for 1,000 ms, a blank screen for 1,000 ms and a noun for 1,000 ms. An interval of 1,000 ms was inserted between trials. Participants received four practice trials before seeing 240 experimental trials presented in three experimental blocks and separated by a short break. The experimental task was 25 min and the entire experiment lasted ⁓90 min.

### EEG recordings and analysis

EEG signals were collected from 64 electrodes secured in an elastic cap and recorded in Neuroscan software. The vertical electrooculogram was captured via two electrodes above and below the left eye. The horizontal electrooculogram was recorded via two electrodes on the left and right external cantus. The left mastoid electrode was used as a reference. The EEG data were re-referenced to the average of both mastoids. All electrode impedances were kept below 5 kΩ. EEG signals were amplified with a band-pass filter between 0.05 and 70 Hz with a sampling rate of 1,000 Hz. The EEGLAB was used for the preprocessing of EEG data. Raw data were down-sampled to 500 Hz and filtered with a high-pass cutoff point of 0.1 Hz and a low-pass cutoff point of 30 Hz. Independent Component Analysis (ICA) using the infomax algorithm (Bell and Sejnowski 1995) was performed to remove vertical or horizontal eye movements, channel noise, muscle artifacts, and 1–3 ICs were removed per participant. Epochs containing amplitudes exceeding ±70 μV were rejected (⁓4.6% of all epochs). Trials with incorrect responses were excluded from analyses (4.7%). The remaining epochs for ERP analysis were on average 110 trials per condition (animate-constraining vs. inanimate-constraining). The EEG was segmented into 3,500 ms epochs that included a 500 ms pre-stimulus display as a baseline, a 1,000 ms classifier display, 1,000 ms interval, and 1,000 ms noun display. Epochs were baseline corrected and the averaged signal in a baseline period was subtracted from the remaining ERPs.

Mean amplitude analyses were performed for pre-noun and after-noun time windows separately. First, mean amplitude analyses for pre-noun time window were performed to examine pre-stimulus predictive brain activity, a negative shift of brain potentials before the onset of the predictable target (i.e. “prediction potential”). To this end, neural activity for animate-constraining versus inanimate-constraining trials for the time window (−200 ms, 0 ms) was analyzed with a baseline correction computed across (−300 ms, −200 ms) before noun onset. We exacted the mean ERP amplitudes at fronto-central electrodes (FC1, FCZ, FC2, C1, CZ, C2, CP1, CPZ, CP2), where the prediction potential is observed (e.g. Grisoni et al. 2017). We investigated whether animate- and inanimate-constraining classifiers can elicit significant negative deflection, by testing pre-noun ERPs against zero, and whether there is difference between animate- versus inanimate-constraining classifiers by directly comparing pre-noun ERPs of two conditions. Second, mean amplitude analyses for after-noun time window were performed to investigate the semantic incongruent effect evoked by the incongruency between classifiers and nouns. Based on previous findings (as reviewed in the Introduction), we expected a N400 effect evoked by incongruent nouns with classifiers. Nine regions of interest (ROIs) were defined to examine the distribution of effects on the scalp. The ROIs include the left-anterior area (F3, F5, FC3), the middle-anterior area (FZ), the right-anterior area (F4, F6, FC4), the left-middle area (C3, C5), the middle–middle area (CZ), the right-middle area (C4, C6), the left-posterior area (P3, P5), the middle-posterior area (PZ), and the right-posterior area (P4, P6). The time window of 300–500 ms was chosen based on visual inspection for N400 effects. Mean amplitudes on this time window were entered into a 2 × 3 × 9 repeated measures analysis of variance (ANOVA) with the factors classifier-type (animate-constraining classifier/inanimate-constraining classifier), congruency condition (Congruent/Incongruent, Animacy-Match/Incongruent, Animacy-Mismatch), and ROIs.

**Fig. 2:**
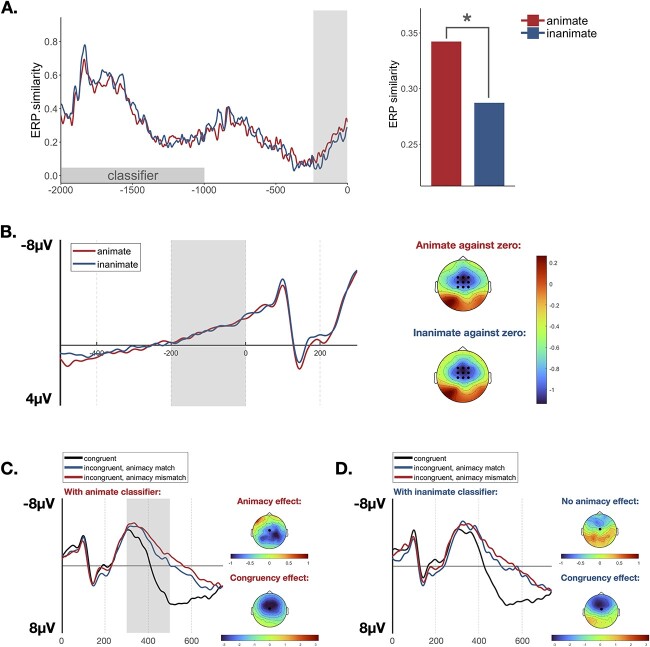
RSA and ERP results. (A) Pre-noun RSA results. It shows similarity values of animate classifier (red line) and inanimate classifier (blue line) before the onset of nouns. The similarity between patterns of neural activity following animate-constraining classifiers was greater than following inanimate-constraining classifiers ⁓240 ms before the onset of nouns. The bar chart presents differences in similarity values from −240 ms to 0 ms between animate- (red bar) versus inanimate- (blue bar) constraining classifiers. (B) Pre-noun ERP waveforms in the animate- (red line) and inanimate- (blue line) constraining classifiers as the average of the fronto-central electrodes (FC1, FCZ, FC2, C1, CZ, C2, CP1, CPZ, CP2). In the last 200 ms before noun onset, both animate- and inanimate-constraining classifiers elicited significant negative-going potentials. (C) and (D) After-noun N400 effects elicited by classifier-noun incongruency at Cz. (C) Shows for the animate-constraining classifiers, additional animacy-mismatch elicits larger N400 in the shaded time window (300–500 ms), revealed by larger N400 by the incongruent and animacy-mismatch condition (red line) than the incongruent and animacy-match condition (blue line). (D) Shows no additional animacy-mismatch effect for inanimate-constraining classifiers (no significant difference between red line and blue line).

RSA analyses were also performed for pre-noun and after-noun time windows separately. First, RSA analyses for pre-noun time window were performed to examine pre-activation of animacy features. We computed an EEG vector across all channels (62 electrodes) and time points by averaging the data for each classifier. For each individual participant, we calculated Pearson’s *r* values to determine the similarity values between the patterns of neural activity following all possible pairs of the animate-constraining classifiers (6*5/2 = 15 pairs) and those of the inanimate-constraining classifiers (6*5/2 = 15 pairs) at each time point from the onset of the classifiers until the onset of nouns. We then averaged these pairwise correlation *r* values to yield averaged similarity values at each time point for each participant for animate- and inanimate-constraining conditions. We then conducted statistical analyses to examine whether the neural similarity values for the animate- versus inanimate- constraining conditions are different *before* the presentation of nouns. To visualize any differences between the two conditions, we averaged these similarity values across all participants at each consecutive time point for animate- and inanimate-constraining conditions. This generated a grand average similarity over time for each condition (see Fig. 2A). Second, RSA analyses for after-noun time window were performed to test whether the neural similarity for the animate nouns is greater than that for the inanimate nouns, as assumed. Each of 12 classifiers was paired with 10 nouns. For each individual participant, we averaged the data of 10 nouns for each classifier, and calculated Pearson’s *r* values to determine the similarity values between the patterns of neural activity following all possible pairs of the animate condition and those of the inanimate condition at each time point during the presentation of nouns. We then averaged these pairwise correlation *r* values to yield averaged similarity values at each time point for each participant for animate and inanimate nouns, and then conducted statistical analyses to examine the difference. For statistical analyses, we conducted a paired *t*-test at each time point across the entire epoch, and critical time windows were identified based on paired *t*-test results that exceeded a preset uncorrected *P*-value threshold of 0.05. For each critical time window, the observed summed *t*-value was determined by summing the individual *t*-values at each time point testing within the time window. We used a nonparametric permutation procedure to protect against problems associated with multiple time points (Maris and Oostenveld 2007). We randomly shuffled the condition labels for each participant, and within each critical time window, we performed *t*-test at each time point and summed individual *t*-values. This procedure was repeated for 1,000 times and formed H0 distribution of summed *t*-values. The observed summed *t*-value falls outside the 95% range is considered to be significant. Separate permutation tests were performed to examine differences for pre-noun and after-noun time windows.

## Results

### Behavioral data

Participants were asked to respond to incongruent phrases, so behavioral data were only available for incongruent phrases. The overall mean response latency for incongruent classifier-noun phrases was 663 ms (standard deviation SD = 195 ms). Response latencies for the Incongruent, Animacy-Mismatch condition (652 ms) were faster than for the Incongruent, Animacy-Match condition (674 ms), suggesting that judgments were made faster for trials with additional animacy-mismatch. A linear mixed-effect model analyses confirmed this observation, showing a significant difference between both conditions (*P* = 0.027, *df* = 118).

### EEG data

We first compared the ERPs of the two conditions to determine if the larger spatial similarities following the animate- versus inanimate-constraining classifiers could be explained by differences in the ERPs evoked by these classifiers. As expected, the ERPs evoked by animate- versus inanimate-constraining classifiers were similar, and a cluster-based permutation test over the entire epoch failed to reveal a significant ERP effect (*P* = 0.359).

Mean amplitude analyses for *pre-noun* time window demonstrate a negative shift of brain potentials before the onset of nouns (i.e. “prediction potential,” see Fig. 2B). In the last 200 ms before noun onset, both animate- and inanimate-constraining classifiers elicited significant negative-going potentials, as documented by *t*-tests against zero (*p*s < 0.001). Animate- and inanimate—constraining classifiers did not elicit difference, as expected (*P* = 0.43). Mean amplitude analyses for *after-noun* time window revealed the semantic incongruent effect evoked by the incongruency between classifiers and nouns. Fig. 2C and D show grand average ERPs for the three congruency conditions at a representative electrode (Cz) under animate classifiers (Fig. 2C) and inanimate classifiers (Fig. 2D). ANOVAs on the mean amplitude of nouns with the factors classifier type, congruency condition, and ROIs revealed main effects of congruency condition (*F* = 38.26, *P* < 0.001) and classifier types (*F* = 24.00, *P* = 0.03), an interaction between the three factors (*F* = 127.66, *P* < 0.001), and a marginally significant interaction between classifier type and congruency condition (*F* = 48.00, *P* = 0.07). The effect of congruency condition revealed that compared with the congruent condition, incongruent conditions elicited a larger negativity in the 300–500 ms time window with a broad distribution in all nine ROIs (*P*-values false discovery rate corrected): left-anterior (*t* = 6.03, *P* < 0.001), left-middle (*t* = 6.47, *P* < 0.001), left-anterior (*t* = 3.98, *P* < 0.001), middle-anterior (*t* = 5.89, *P* < 0.001), middle–middle (*t* = 6.14, *P* < 0.001), middle-posterior (*t* = 3.69, *P* = 0.001), right-posterior (*t* = 7.03, *P* < 0.001), right-middle (*t* = 6.61, *P* < 0.001), and right-posterior (*t* = 4.74, *P* < 0.001). Following the interactions, separate analyses were conducted for each classifier type. For animate classifiers, there was a graded effect with the largest negativity in the Incongruent, Animacy-Mismatch condition, compared to the Incongruent, Animacy-Match condition, due to the additional mismatch in animacy. Pairwise comparisons for each ROI revealed the additional mismatch effect in animacy with a broad distribution, i.e. left-posterior (*t* = 2.55, *P* = 0.026), middle-middle (*t* = 2.59, *P* = 0.016), middle-posterior (*t* = 2.38, *P* = 0.038), right-middle (*t* = 2.24, *P* = 0.035), and right-posterior (*t* = 3.11, *P* = 0.007). Contrary to animate classifiers, for inanimate classifiers the additional mismatch effect from animacy disappeared in all nine ROIs (*t*s < 1.52; *p*s > 0.14).

Grand average similarity waveforms over time are displayed in Fig. 2A for animate- versus inanimate-constraining classifiers. Statistical analyses revealed that during the presentation of classifiers (−2,000 ms to –1,000 ms), there was no greater neural similarity for animate-constraining classifiers, relative to inanimate-constraining classifiers. The neural similarity within the animate-constraining classifier group was greater than that of the inanimate-constraining condition, from 240 ms before the onset of noun. Statistical analyses confirmed that the greater neural similarity within animate-constraining classifier group was observed before noun onset, relative to neural similarity within the inanimate-constraining condition (−240–0 ms; *P* = 0.036), reflecting the prediction of animacy features associated with the upcoming words. RSA analyses for after-noun time window revealed that the patterns of brain activity were more similar among animate nouns than among inanimate nouns throughout the whole time window of noun presentation (*P* = 0.04). To test for the possibility that word frequency would explain the RSA effects, we used linear mixed-effects models to predict the neural similarity value in the predictive time window with type of animacy constraint and word frequency. We measured frequency similarity by taking the absolute value of the difference of word frequency between classifiers, and included this measure in the model. The maximal model included random intercepts for participants and items, and participant random slopes for type of animacy constraint and frequency similarity. Because *type of animacy constraint* were manipulated between items, item slope adjustments were not specified for the factor. In cases in which the maximal model failed to converge, we sequentially simplified the random effects until convergence was achieved. The mixed-effects model analysis was performed using the “lme4” package as implemented in R. Significance of fixed effects of the model was assessed with the *anova()* function from package *lmerTest,* using the Satterthwaite method of approximation for degrees of freedom. Results revealed that the classifier type (animate vs. inanimate) remained a significant effect (*F* = 3.20, *P* = 0.048), even with word frequency included. The effect of frequency failed to reach significance (*F* < 1).

## Discussion

In the present study, RSA on EEG data was conducted to investigate whether animacy features would be pre-activated without a highly constraining context. Chinese classifiers were varied in animacy constraint (animal-constraining and inanimate-constraining) and were followed by Congruent, Incongruent but animacy-matched, or Incongruent but animacy-mismatched nouns. Behavioral response latencies showed a faster response (22 ms) for the Incongruent, Animacy-Mismatch condition than the Incongruent, Animacy-Matched condition, due to additional animacy violation. N400 effect was observed for incongruent conditions, with larger negativity for an additional mismatch in animacy (only for animate-constraining classifiers). Both animate- and inanimate-constraining classifiers elicited a negative shift of brain potentials before the onset of nouns, which is likely to reflect a prediction potential. Of greatest relevance to the study was the RSA result, i.e. greater similarity among patterns of neural activity following the animate-constraining than following the inanimate-constraining classifiers. The similarity effects were significant from ~240 ms before the onset of nouns, which suggests the pre-activation of semantic features of following nouns.

### The ERP effects of semantic and animacy violations of nouns

The present study manipulated two types of violation between classifiers and following nouns. For the semantic violation, nouns were semantically incongruent with the preceding classifiers. For the animacy mismatch, there were additional conflict of animacy (the Incongruent, Animacy-Mismatch condition), or no conflict of animacy (the Incongruent, Animacy-Match condition). Semantic violation elicited N400 effects, which is compatible with previous findings, reflecting difficulty in semantic integration (e.g. Federmeier and Kutas 1999; Zhang et al. 2012; Zhou et al. 2010). Moreover, the additional mismatch in animacy resulted in a further increase in N400 amplitude, suggesting that animacy information of classifiers and nouns is processed during classifier-noun phrases. Interestingly, this additional animacy-mismatch effect only emerged for animate-constraining classifiers, but not for inanimate-constraining classifiers. The null effect of animacy-mismatch for inanimate classifiers is in line with the results from the closely matched counterpart by Zhang et al. (2012) where only inanimate classifiers were included, and no additional animacy-mismatch effect was observed. Our study extended the investigation of animacy-mismatch to animate classifiers and observed the animacy-mismatch effect. The divergence in animacy-mismatch effect between animate versus inanimate classifiers may reflect the possibility that animate classifiers are more constraining for animacy relative to inanimate classifiers.

### The classifier-driven prediction of animacy features of upcoming nouns

Recently, a negative-going potential shift starting hundreds of milliseconds prior to predictable stimuli has been highlighted as a neurophysiological index of prediction (e.g. Pulvermüller and Grisoni 2020). The cortical sources underlying the prediction potential reflect specific perceptual and semantic features of anticipated stimuli before predictable stimuli appear (not before unpredictable ones), which suggests its predictive nature (Grisoni et al. 2016, 2017, 2019, 2022). In the present study, animate- and inanimate-constraining classifiers elicited such brain potentials before the onset of nouns, which is likely related to prediction.

More critically, the present study reveals neural similarity effects before the presentation of nouns, which provides more direct evidence for the pre-activation of coarse-grained semantic features that distinguish between upcoming animate and inanimate items. Our results cannot be explained by lexical-semantic processing of the classifiers across conditions because they were matched well in semantic similarity structure and other linguistic properties. In addition, the animacy RSA effect was not found during the presentation of classifiers and was present only after classifiers and before the onset of nouns, which strongly suggests that it was not elicited by processing of classifiers. Moreover, the critical assumption of the present study is that animate nouns share more common semantic features than inanimate nouns, and thus animate nouns are more similar in neural activity patterns than inanimate nouns. It is important to confirm this assumption. In the present study, the mean semantic similarity within the animate nouns was indeed greater than within the inanimate nouns, and correspondingly animate nouns elicited greater neural similarity than inanimate nouns.

As reviewed in Introduction, previous investigations have mainly focused on the pre-activation of specific lexical words. However, in natural language, most contexts cannot constrain a specific lexical item, but likely constrain semantic features. Therefore, it is theoretically important that we verified that comprehenders can predict semantic information of upcoming words beyond individual words. This finding aligns with Wang et al. (2020) in which participants hear three-sentence context, followed by animate-constraining or inanimate-constraining verbs, and show more similar patterns of neural activity following animate-constraining verbs than following inanimate-constraining verbs. One caveat with this type of design is that neural similarity may reflect not the similarity in the meanings of the predicted words but rather the similarity of the meanings of the preceding context. To minimize the potential possibility, in the present study, we reduced prior context and only used a single classifier to constrain animate or inanimate nouns, which can provide clear evidence for the pre-activation of semantic features.

In sum, we provide neural evidence for the prediction of coarse-grained animacy-related semantic features driven by isolated Chinese classifiers.

## Data Availability

For protection of participants’privacy, the data are available upon request to the authors. Please email the corresponding author for more information.
